# Modified technique of closing the port site after multiport thoracoscopic surgery using the shingled suture technique: a single centre experience

**DOI:** 10.1186/s12893-021-01220-4

**Published:** 2021-04-30

**Authors:** Haitao Xu, Shuai Ren, Tianyu She, Jingyu Zhang, Lianguo Zhang, Teng Jia, Qingguang Zhang

**Affiliations:** Department of Thoracic Surgery, Binzhou Medical University Hospital, No. 661 Huanghe 2nd Road, Binzhou, 256603 Shandong People’s Republic of China

**Keywords:** Multiport, Video‐assisted thoracoscopic surgery, Chest tube, Suture

## Abstract

**Background:**

Due to improvements in operative techniques and medical equipment, video-assisted thoracoscopic surgery has become a mainstay of thoracic surgery. Nevertheless, in multiport thoracoscopic surgery, there have been no substantial advances related to the improvement of the esthetics of the site of the chest tube kept for postoperative drainage of intrathoracic fluid and decompression of air leak after thoracoscopic surgery. Leakage of fluid and air around the site of the chest tube can be extremely bothersome to patients.

**Methods:**

From March 2019 to April 2020, we used a modified technique of closing the port site in 67 patients and the traditional method in 51 patients undergoing multiport thoracoscopic surgery due to lung disease or mediastinal disease. We recorded patients’ age, gender, body mass index, surgical method, postoperative drainage time, and postoperative complications.The NRS pain scale was used to score the pain in each patient on the day of extubation.The PSAS and the OSAS were used for the assessment of scars one month after surgery.

**Results:**

In the modified technique group, only one patient (1.49%) had pleural effusion leakage, compared with five patients (9.80%) in the traditional method group (*P* < 0.05). There were no significant differences in the pain of extubating and wound dehiscence between the two groups. However,the incidence rates of wound dehiscence in the modified technique group were lower than in the traditional method group. There were no post-removal pneumothorax and wound infection in either of the groups. Significant differences in the PSAS and OSAS were observed between the groups,where the modified technique group was superior to the traditional method group.

**Conclusions:**

The modified technique of port site closure is a leak-proof method of fixation of the chest tube after multiport thoracoscopic surgery. Moreover, it is effective and preserves the esthetic appearance of the skin.

## Background

There are different approaches to video-assisted thoracoscopic surgery, such as multiport and uniport approaches. Since Gonzales first reported uniport thoracoscopy for a lobectomy in 2011 [1], different operative procedures [2, 3] and techniques for fixation of postoperative drainage tubes [4, 5] have attracted attention. Nevertheless, in multiport thoracoscopic surgery, there have been no substantial improvements in the esthetic management of the port site or in the optimal means of assuring an adequate watertight fixation of the chest tube to prevent leakage of fluid or air around the postoperative drainage tube. Here, we shared our experience using a new method of thoracoscopy port site closure that improves both the esthetic appearance of thoracoscopy port site and the fixation and function of the postoperative chest tube.

## Methods

### Patients

We retrospectively analyzed patients with lung disease or mediastinal disease who had undergone multiport thoracoscopic surgery in the Binzhou Medical University Hospital between March 2019 and April 2020. A total of 67 patients received a modified technique of closing the port site using the shingled suture technique (the modified technique group) by the team of Haitao Xu. A total of 51 patients received the traditional method of fixation of the chest tube using two nonabsorbable sutures to close the skin on each side of the drainage tube (the traditional method group) by another team. We recorded patients’ age, gender, body mass index (BMI), surgical method, postoperative drainage time, and postoperative complications. Postoperative complications included pleural effusion leakage, post-removal pneumothorax, wound infection, and wound dehiscence. The Numeric rating scale [6] (NRS) pain scale was used to score the pain in each patient on the day of extubation. The Patient Scar Assessment Scale [7] (PSAS) and the Observer Scar Assessment Scale [7] (OSAS) were used for the assessment of scars one month after surgery. This study was approved by the Ethics Committee of the Binzhou Medical University Hospital.

### Surgical technique

For multiport thoracoscopy, the thoracoscopic incision was about 15 mm long and was made in the seventh or eighth intercostal space at the mid-axillary line. A 12 mm Trocar (Ethicon, Somerville, NJ, US) was used to establish a path for the thoracoscopy camera. A second working port site incision (30 mm) was made in the fourth or fifth intercostal space at the anterior axillary line, and a third working port site incision (20 mm) was made in the eighth intercostal space at the scapular line. After finishing the intrathoracic aspect of the operation, a chest tube was inserted through the smallest incision for postoperative evacuation of any accumulated intrathoracic fluid and decompression of any air leak. For our technique, we divided the thoracoscopy port site incision where the chest tube is to exit into three zones: T_C_ was the region located in the center of the site of the drainage tube, while T_L_ and T_R_ were the regions to be closed located at both sides of the chest tube.

**Fig. 1:**
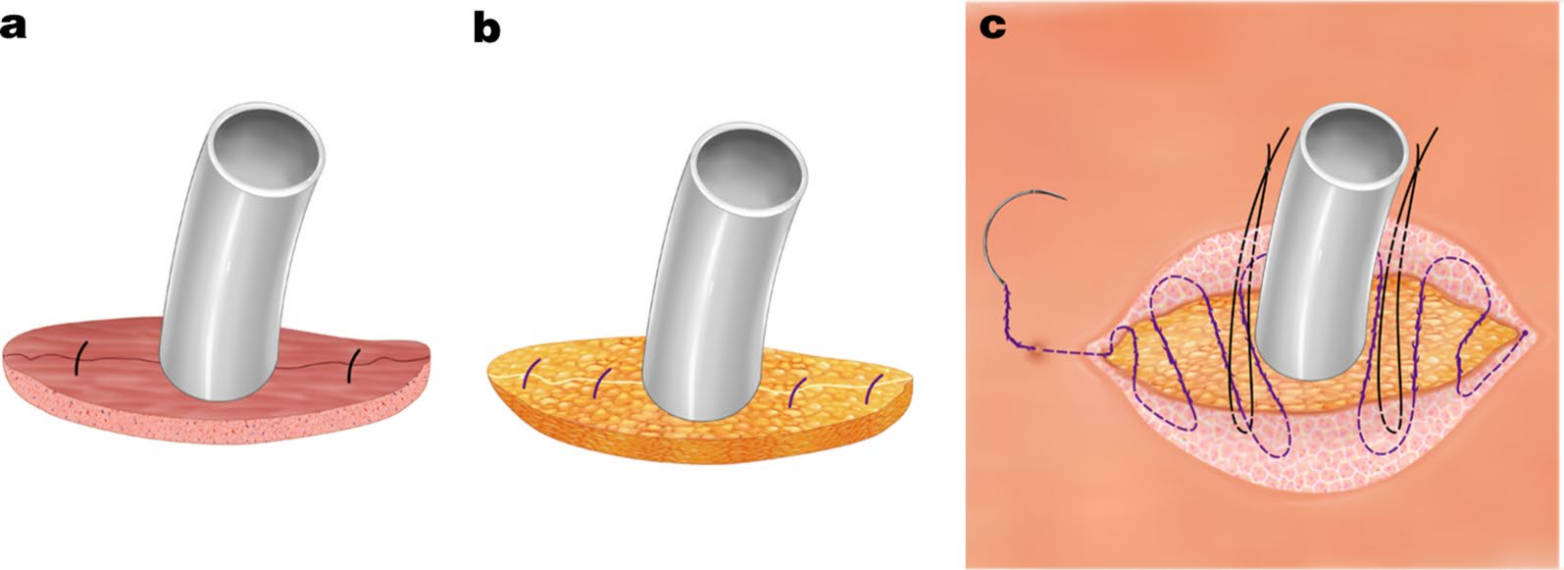
**a** Intermittent suture of the deep muscles of the port site. **b** Intermittent suture of the subcutaneous adipose tissue of the port site. **c** Removal-free, absorbable, continuous intradermal suture of the skin

Before inserting the chest tube, two separate 2-0 Vicryl sutures (Ethicon, Somerville, NJ, US) were used to approximate the deep muscle layers with their investing fascia, one on each side (the midpoints of T_L_ and T_R_); however, after the sutures were placed, they were not tied at this point, but the ends were exteriorized out the incision to be tied later. After the chest tube was inserted, the sutures were tied to approximate the muscle layer (Fig. 1a). Because the port site opening was narrow, this approach facilitated placing the sutures for optimal closure of the muscle layer without the chest tube interfering with the placement of the sutures, which ensured a tight closure of this deep space.

Next, four separate 1 -0 Vicryl sutures (Ethicon, Somerville, NJ, US) were used for intermittent suture of the subcutaneous adipose tissue at 1/4 and 3/4 of the distance between T_L_ and T_R_ to ensure an eventual tight closure of the subcutaneous tissue of the port site around the exiting chest tube (Fig. 1b).

**Fig. 2:**
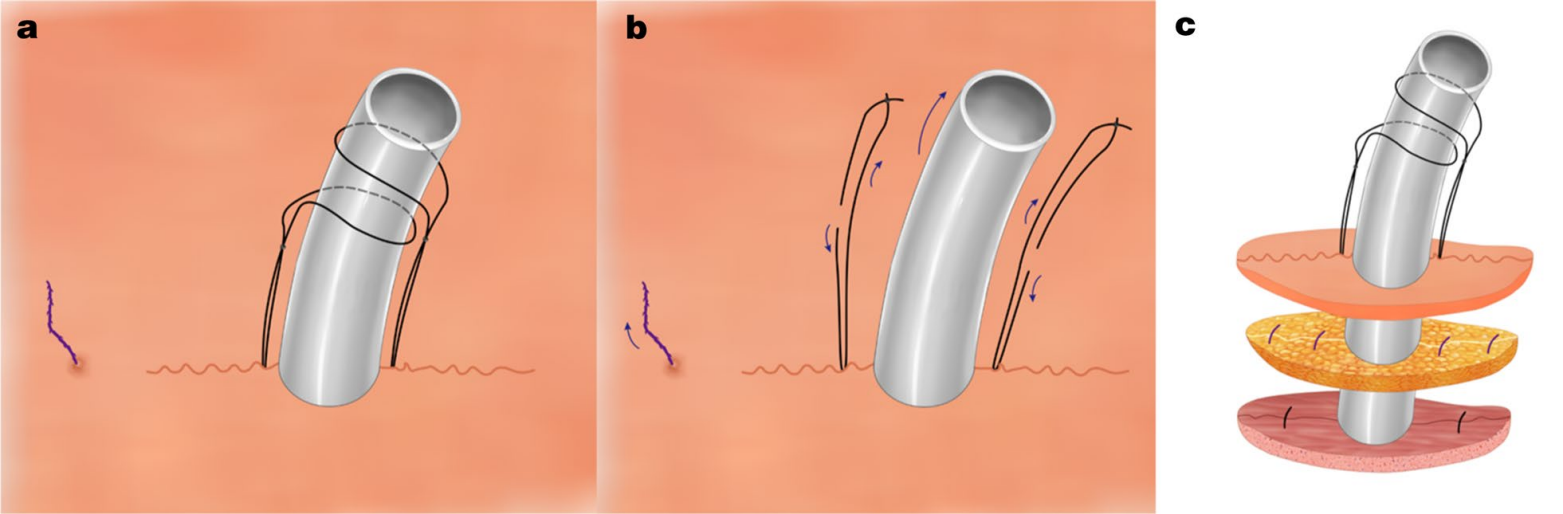
**a** Fixation of the chest tube and skin closure. **b** Removal of the chest tube. **c** Shingled suture technique

Next, two separate 1-0 silk braided nonabsorbable sutures (Ethicon, Somerville, NJ, US) were sewn into subcutaneous tissues near the chest tube, one on each side, and tied about 3–5 cm above the skin around the chest tube to fix the chest tube securely. Again, these sutures for tube fixation were not tied in the subcutaneous tissues. Finally, a 3-0 unidirectional, barbed, absorbable suture (Angiotech, Aguadilla, Puerto Rico) was used for a continuous intradermal suture closure of the port site skin. The suture went around one side of the chest tube with the 1-0 silk braided sutures positioned between the chest tube and the intradermal suture. Several centimeters of the ends of the intradermal suture were left outside the incision at one end, and the end of the suture was pulled to tighten the intradermal wound closure (Figs. 1c and 2a). These intradermal sutures did not need to be removed and would be resorbed over the following few weeks. This approach led to a more scarless result.

When the time was right to remove the chest tube, the incision was disinfected, and the two separate 1-0 silk braided sutures used for fixing the drainage tube were cut at the points of their fixation to the chest tube and removed from the subcutaneous tissues. Then, multiple layers of sterile gauze were used to cover the site of the drainage tube, patients were instructed to inhale deeply and to hold breath, and while applying pressure on the sterile gauze, the chest tube was removed rapidly. Next, the end of the unidirectional barbed suture was tightened to close the skin incision, and the suture was cut near the end of the incision (Fig. 2b).We call this approach a “shingled suture technique” (Fig. 2c).

### Statistical analysis

SPSS 22.0 statistical software was used for data analysis.The measurement data were analyzed by independent sample *t*-test and expressed in the form of mean ± standard deviation. The counting data were compared by the chi-square test or Fisher’s test. A value of *P* < 0.05 was considered statistically significant.

## Results

**Fig. 3:**
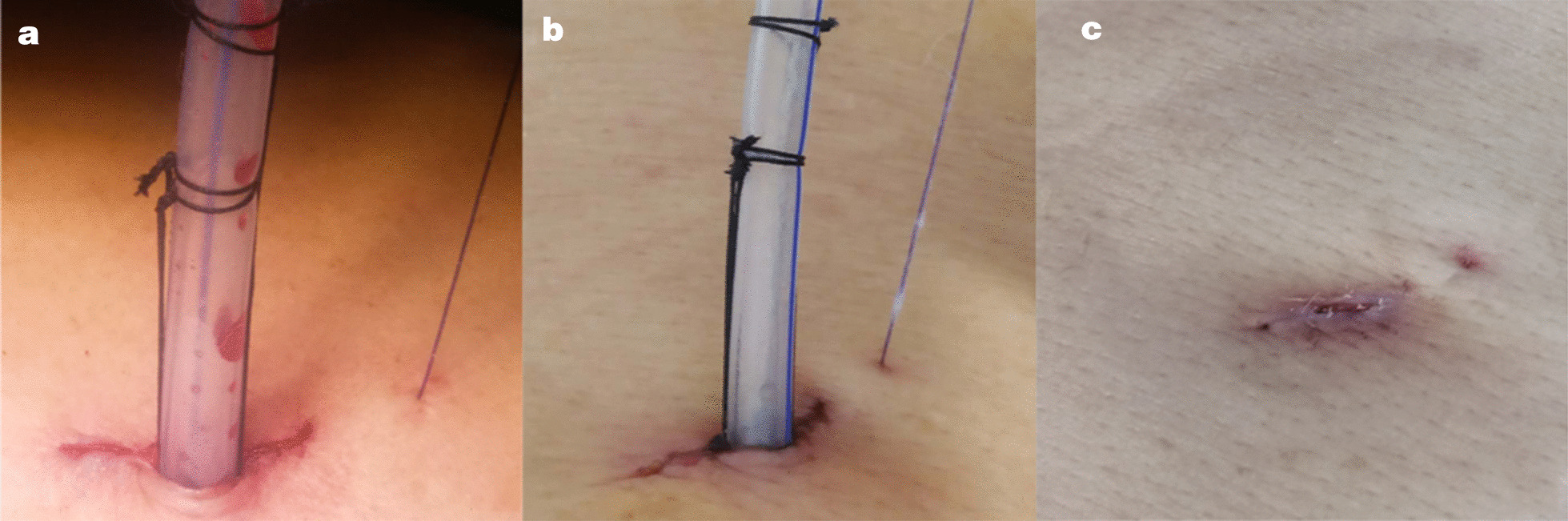
Closing the port site after operation using the shingled suture technique. **a** Day of operation. **b** 3 days after the operation. **c** 12 days after the operation

The clinical characteristics of the patients are shown in Table 1. There were no significant differences in clinical features between the two groups (*P >* 0.05). The chest tubes were removed 2–13 days after the thoracoscopic procedure. Table 2 shows a comparison of the postoperative complications between the two groups. There was a significant difference in the rate of pleural effusion leakage between the two groups (*P* = 0.04). Specifically, the modified technique group was superior to the traditional method group(1.49 % vs. 9.80 %). Two (3.92 %) of the patients in the traditional method group had wound dehiscence, while there was no wound dehiscence in the modified technique group (*P =* 0.10). There were no post-removal pneumothorax and wound infection in either of the groups. No significant difference in the pain of extubating was observed between the two groups (*P* = 0.49), which shows that the pain was tolerable for patients and that our new approach did not entail additional pain. There were significant inter-group differences in the PSAS (*P* = 0.002) and OSAS (*P* = 0.001), the modified technique group was superior to the traditional method group for both scores. We used the modified technique (Fig. 3a, b and c) in 67 patients, and most of the patients were satisfied with the healing of their thoracoscopic incision.

**Table 1:** Clinical characteristics of patients

Characteristics	Modified technique group (n = 67) n (%)	Traditional method group (n = 51) n (%)	*F/χ* ^2^ value	*P* value
Age,years			0.21	0.64
Mean ± SD	57.27 ± 12.73	56.96 ± 11.75		
Range	16–77	20–79		
Gender			1.66	0.19
Male	41 (61.2)	37 (72.5)		
Female	26 (38.8)	14 (27.5)		
BMI,kg/m^2^			2.84	0.09
Mean ± SD	1.72 ± 0.15	1.78 ± 0.12		
Range	1.38–1.99	1.52–2.04		
Surgical method			2.21	0.33
Segmentecomy	4 (6.0)	7 (13.7)		
Lobectomy	56 (83.6)	38 (74.5)		
Mediastinal mass resection	7 (10.4)	6 (11.8)		
Postoperative drainage time,days			0.91	0.34
Mean ± SD	5.10 ± 2.37	5.43 ± 2.55		
Range	2–13	2–12

**Table 2:** Comparison of postoperative complications between two groups

Postoperative complications	Modified technique group (n = 67) n(%)	Traditional method group (n = 51) n (%)	* X* ^2^/*F* value	*P* value
Pleural effusion leakage	1 (1.49)	5 (9.80)	4.15	0.04
Post-removal pneumothorax	0	0	−	−
Wound infection	0	0	−	−
Wound dehiscence	0	2 (3.92)	2.67	0.10
NRS				
Mean ± SD	2.12 ± 1.45	1.78 ± 1.15	0.48	0.49
PSAS				
Mean ± SD	6.88 ± 2.10	7.92 ± 2.83	10.01	0.002
OSAS				
Mean ± SD	5.76 ± 1.74	6.80 ± 2.71	11.62	0.001

## Discussion

Video-assisted thoracoscopic surgery has recently gained attention as an alternative surgical option for conventional open surgery because of its advantages in reducing postoperative pain and chest wall paresthesia and its association with better outcomes  [8, 9].

**Fig. 4:**
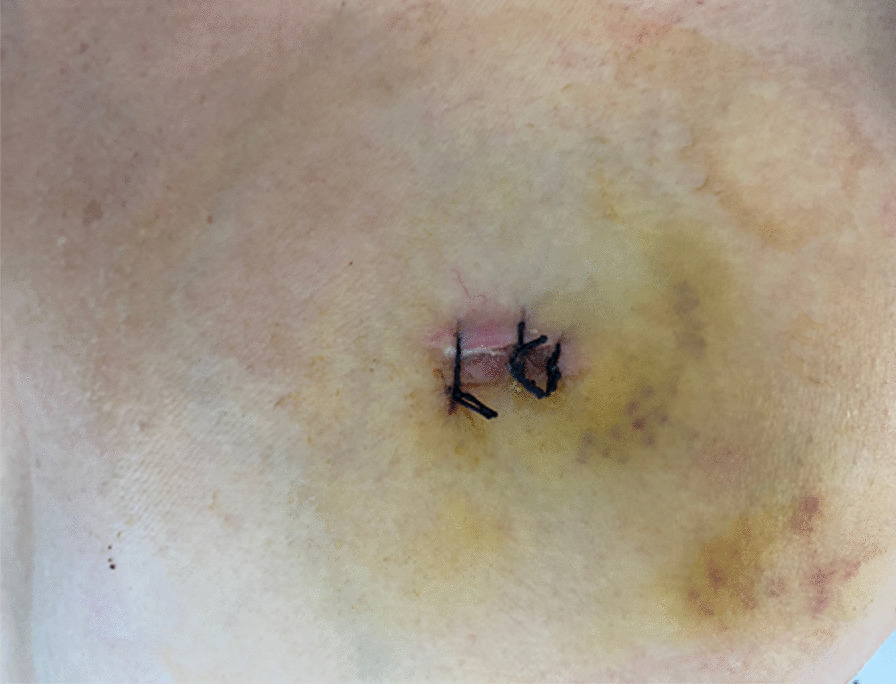
Traditional method, post-removal chest tube

In multiport thoracoscopic surgery, most surgeons in our centre or country use the traditional method of fixation of the chest tube with at least two nonabsorbable sutures to close the skin on each side of the drainage tube, or with three sutures, where the last one is left without a knot, which is used for the closure of the port site after extubation. The purpose of the latter technique is to close the port site to prevent postoperative leakage of fluid or air. However,even then, drainage of fluid or air leaking around the tube often occurs, making the drainage site difficult to manage and tending to affect the ultimate esthetics of the incision site (Fig. 4). The patients’ point of view of the cosmetic appearance is also important, considering that the extent of scarring affects their self-assessment of the treatment outcome [10, 11].

Using our modified technique, the site of the deep of the muscle layer and the closure of the adipose layers overlap. In addition, the use of the unidirectional intradermal barbed suture [12, 13] allows us to suture the skin closure tightly around the chest tube at the end of the operation. Due to similarity to shingling the roof, we named this a shingled suture. This technique decreases extravasation of fluid and air leakage around the chest tube. In addition, at the time of removal of the chest tube, the unidirectional aspect of the barbed intradermal suture allows us to further tighten and close the defect in the port site left after the removal of the chest tube to increase the esthetics of the site without the need to remove the absorbable intradermal suture.

The modified technique requires conducting three different steps, which takes about 6–8 min, while the traditional method only requires intermittent suture, which takes about 2–3 min. Although the modified technique is more time-consuming compared with the traditional method, it is superior in terms of drain-related complications and cosmetic outcomes. Moreover, the modified technique does not entail additional pain. Since this is a retrospective study, we acknowledge that it has some limitations, including the retrospective design, lack of consideration of factors affecting wound healing, and recording of the time duration for each suture. In this study, we analysis and comparison with existing data suggested that our modified technique is safe and effective with a good cosmetic outcome. However, there is still limited clinical experience with the modified method, and further studies are warranted.

This technique of port site closure minimized leakage of fluid around the drainage tube and also led to excellent results in terms of the esthetics of the port site wound.This suture is removal-free,which can also decrease doctors’ work burden and improve patients’ medical experience.

## Conclusions

In summary, the modified technique of port site closure is a leak-proof method of fixation of the chest tube after multiport thoracoscopic surgery, which provides good effects with esthetic appearance of the skin.

## Data Availability

The data that support the findings of this study are available from the corresponding author on reasonable request. Emails could be sent to the address below to obtain the shared data: bzmcxht@163.com.

## Abbreviations

BMI: Body mass index
SD: Standard deviation
NRS: Numeric rating scale
PSAS: Patient scar assessment scale
OSAS: Observer scar assessment scale
